# Supplementary material for: Replication of Missense OTOG Gene Variants in a Brazilian Patient with Menière’s Disease
Source: Genes (Basel). 2025 May 28;16(6):654. doi: 10.3390/genes16060654 (PMC12193698; doi:10.3390/genes16060654)
Supplement: Supplementary file 1 [file genes-16-00654-s001.zip › genes-3657012-supplementary.pdf]

Replication of missense OTOG gene variants in a Brazilian cohort of Menière's Disease

Giselle Bianco-Bortoletto[1,2,3]; Geovana Almeida Carneiro[4]; Helena Fabbri-Scallet[2,5]; Alberto M. Parra-Perez[1,6,7]; Karen de Carvalho Lopes[8]; Tatiana de Almeida Lima Sá Vieira[8]; Fernando Ganança[8]; Juan Carlos Amor-Dorado[9]; Andres Soto-Varela[10,11,12]; Jose A. Lopez-Escamez[1,6,7]*; Edi Lucia Sartorato[2,3]*.

[1]Meniere Disease Neuroscience Research Program, Faculty of Medicine & Health, School of Medical Sciences, The Kolling Institute, University of Sydney, Sydney, New South Wales, Australia.

[2]Laboratory of Human Molecular Genetics, Center for Molecular Biology and Genetic Engineering-CBMEG, State University of Campinas-UNICAMP, Sao Paulo, Brazil.

[3]Programa de Pós-Graduação em Ciências Médicas, Faculty of Medical Sciences, State University of Campinas-UNICAMP, Sao Paulo, Brazil.

[4]Department of Biochemistry and Tissue Biology, Laboratory of Paracrine Signalling in Tissue Organisation-SPOT, Programa de Pós-Graduação em Biologia Molecular e Morfofuncional, Biology Institute, State University of Campinas-UNICAMP, Sao Paulo, Brazil.

[5]Postdoctoral Researcher Program, Faculty of Medical Sciences, State University of Campinas, Sao Paulo, Brazil.

[6]Otology & Neurotology Group CTS495, Division of Otolaryngology, Department of Surgery, Instituto de Investigación Biosanitaria, ibs.GRANADA, Granada, Universidad de Granada, Granada, Spain

[7]Sensorineural Pathology Programme, Centro de Investigación Biomédica en Red en Enfermedades Raras, CIBERER, Madrid, Spain

[8]Otolaryngology and Head and Neck Surgery Department of Federal University of São Paulo, Brazil.

[9]Department of Otolaryngology, Hospital Can Misses, Ibiza, Spain.

[10]Division of Neurotology, Department of Otorhinolaryngology, Complexo Hospitalario Universitario, Santiago de Compostela, Spain.

[11]Department of Surgery and Medical-Surgical Specialities, Universidade de Santiago de Compostela, Santiago de Compostela, Spain.

[12]Health Research Institute of Santiago (IDIS), Santiago de Compostela, Spain.

*Corresponding authors:

Jose A. Lopez-Escamez, Meniere's Disease Neuroscience Research Program, Faculty of Medicine & Health, School of Medical Sciences, The Kolling Institute, University of Sydney, Sydney, New South Wales, Australia. Email: jose.lopezescamez@sydney.edu.au

Edi Lucia Sartorato, Laboratory of Human Genetics, Center for Molecular Biology and Genetic Engineering-CBMEG, Universidade Estadual de Campinas-UNICAMP, Campinas, São Paulo, Brazil. Email: sartor@unicamp.br

Supplementary Figures

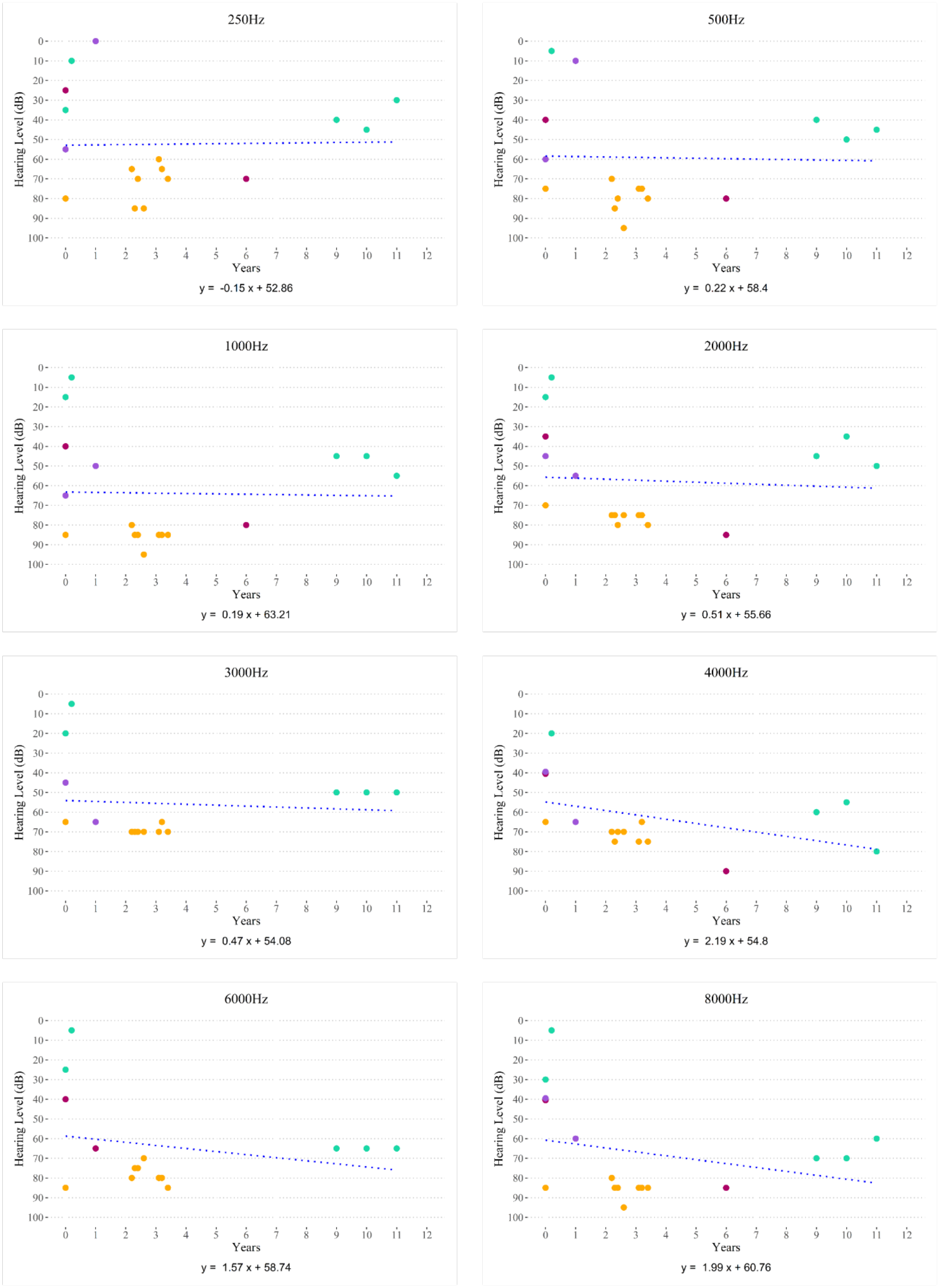

Supplementary Figure S1. Scattered plot showing hearing thresholds and the duration of the disease for each frequency in the four patients carrying the chr11:17599671C>T variant in OTOG gene. Green dots represent the SMD Brazilian proband; Magenta and Violet represent the two SMD Spanish patients;

Yellow dots represent the FMD Spanish patient; Regression equation is represented by the blue dotted lines. The worst ear data was used for the plot. There were no available data for the SMD Spanish patients at 3kHz (magenta dot) and 6kHz (violet dot).

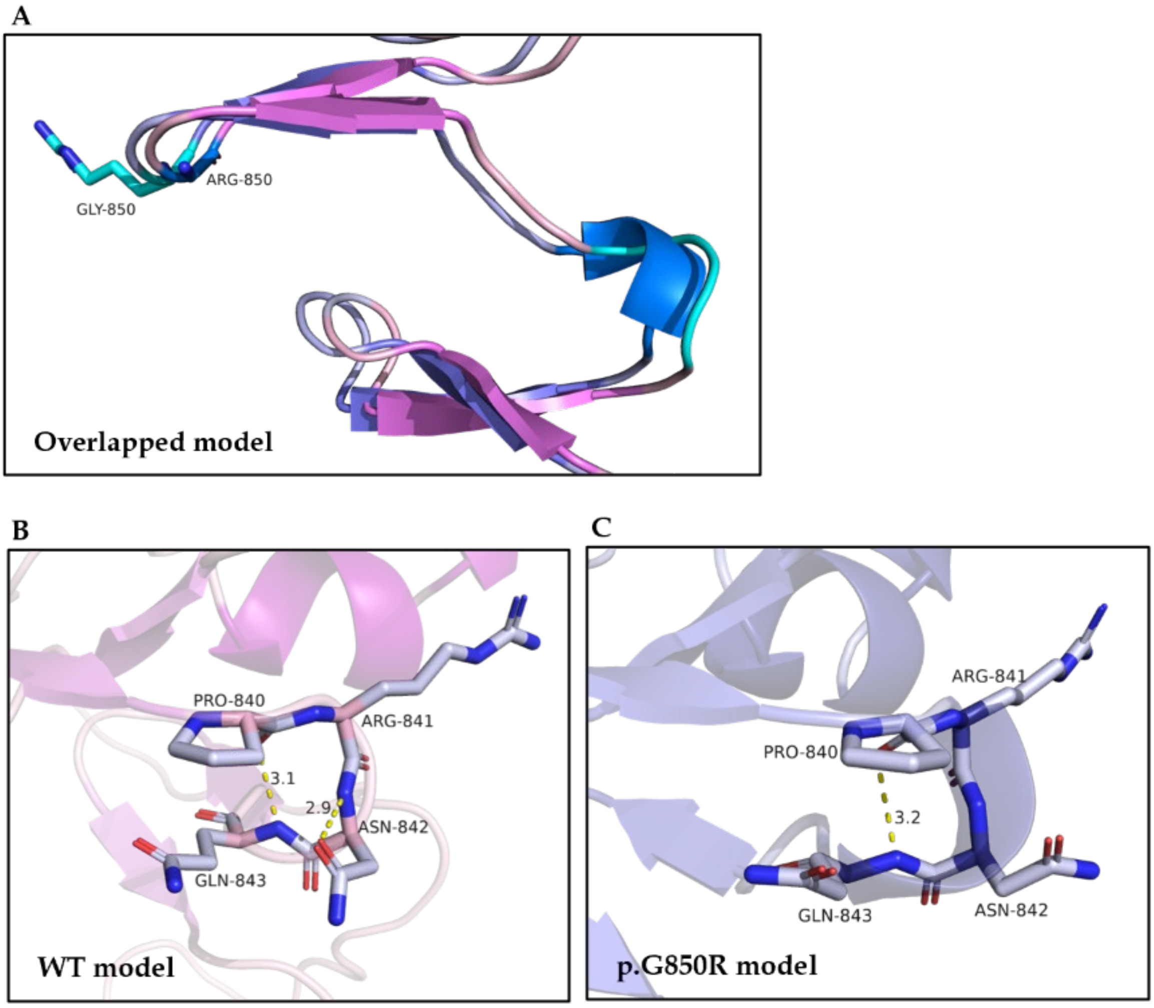

Supplementary Figure S2. Changes in Otogelin 3D structure. Polar interactions between amino acids are represented by yellow dotted lines, with respective distances measured in Ängstrom. (a) Wild type and mutated Otogelin structures overlapped, showing the creation of a α-helix at p.841-843. (b) Wild type Otogelin amino acids arrange and polar contacts at p.840-843. (c) Mutated Otogelin amino acids arrange and polar contacts at p.840-843. The polar contact formed within p.Asn842 in the WT structure no longer exists in the mutated one.

Supplementary Tables

Supplementary Table S1. Coefficient of determination R2 and p value for each scattered plot.

Frequency (Hz)	p-value	R^2
250	0.9356	0.00045
500	0.9092	0.000896
1000	0.9246	0.000617
2000	0.7729	0.005722
3000	0.7588	0.007508
4000	0.0773	0.1934
6000	0.9356	0.00045
8000	0.2674	0.08128

Supplementary Table S2. Model quality validation of predicted structural models for selected variants in Otogelin.

Variant Modelled	Amino acid change	Modelling method	Software used	Evaluation method				
				Dope score	molpdf score	GA341 score	ERRAT score	Ramachandran plot
chr11:17599671C>T	p.Pro1240Leu	Homology	Modeller v10.5	-247147.0625	18634.0332	1.00000	75.6295	91.9% core
chr11:17576581G>T	p.Gly850Arg	Homology	Modeller v10.5	-247374.6719	18558.66992	1.00000	72.9905	91.7% core
chr11:17594108 C>T	p.Pro1129Leu	Homology	Modeller v10.5	-247354.5625	18280.60742	1.00000	73.7158	91.7% core
chr11:17599671C>T + chr11:17576581G>T + chr11:17594108 C>T	p.Pro1240Leu + p.Gly850Arg + p.Pro1129Leu	Homology	Modeller v10.5	-246513.3281	18042.59961	1.00000	72.2502	91.2% core

Supplementary Table S3.

Human Splicing Finder PRO predictions for chr11:17599671C>T variant in *OTOG* gene.

Regulatory elements	Name	Position	Sequence	Status
Exonic Splicing Enhancer (ESE)	ESE_SRp40	chr11:17599665	TTTCAGC	Site Broken
Exonic Splicing Silencer (ESS)	IIE	chr11:17599666	TTCAGT	Site Created
Exonic Splicing Enhancer (ESE)	EIE	chr11:17599667	TCAGTG	Site Created
Exonic Splicing Silencer (ESS)	IIE	chr11:17599667	TCAGTG	Site Created
Exonic Splicing Silencer (ESS)	IIE	chr11:17599668	CAGTGT	Site Created
Exonic Splicing Enhancer (ESE)	ESE_ASF	chr11:17599668	CAGCGTA	Site Broken
Exonic Splicing Enhancer (ESE)	ESE_ASFB	chr11:17599668	CAGCGTA	Site Broken
Exonic Splicing Enhancer (ESE)	ESE_SRp55	chr11:17599669	AGCGTA	Site Broken
Exonic Splicing Silencer (ESS)	IIE	chr11:17599669	AGTGTA	Site Created
Exonic Splicing Silencer (ESS)	Fas ESS	chr11:17599669	AGTGTA	Site Created
Exonic Splicing Silencer (ESS)	IIE	chr11:17599670	GTGTAT	Site Created
Exonic Splicing Enhancer (ESE)	RESCUE ESE	chr11:17599671	CGTATG	Site Broken
Exonic Splicing Enhancer (ESE)	EIE	chr11:17599671	TGTATG	Site Created
Exonic Splicing Silencer (ESS)	IIE	chr11:17599671	TGTATG	Site Created
